# Neuroprotective synergy of curcumin and *Glycyrrhiza glabra* in an alzheimer’s model: implications for inflammation and redox pathways

**DOI:** 10.3389/fphar.2025.1661706

**Published:** 2025-10-14

**Authors:** Shanshan Liu, Jing Yan, Jian Dong

**Affiliations:** ^1^ Department of Neurology, Union Jiangbei Hospital, Huazhong University of Science and Technology, Wuhan, Hubei, China; ^2^ Department of Respiratory and Critical Care Medicine, Union Jiangbei Hospital, Huazhong University of Science and Technology, Wuhan, Hubei, China

**Keywords:** Alzheimer's disease, curcumin, Glycyrrhiza glabra, neuroinflammation, oxidative stress, cognitive dysfunction

## Abstract

**Objectives:**

Alzheimer’s disease (AD) is a progressive neurodegenerative disorder marked by cognitive impairment, amyloid-beta (Aβ) accumulation, tau hyperphosphorylation, and chronic neuroinflammation. Despite symptomatic treatments, no current therapies halt disease progression. Traditional Chinese Medicine (TCM) offers potential multi-targeted interventions in complex diseases like AD. In this study, we evaluated the individual and combined neuroprotective effects of curcumin and Glycyrrhiza glabra in a D-galactose sodium nitrite-induced mouse model of AD. Behavioral analysis, biochemical assays, and molecular profiling were conducted to assess cognitive function, inflammatory cytokine expression, oxidative stress, and AD-related protein levels.

**Methods:**

Mice were randomly assigned to five groups: control, AD model, curcumin-treated, *Glycyrrhiza glabra*-treated, and combination-treated groups. Cognitive function was assessed using the Morris water maze. Levels of neuroinflammatory cytokines interleukin-6(IL-6) and tumor necrosis factor-alpha (TNF-α), AD-related proteins amyloid precursor protein (APP) and microtubule-associated protein tau (MAPT),oxidative stress (MDA), and antioxidant capacity (SOD) were evaluated via quantitative PCR and Western blotting.

**Results:**

Curcumin alone modestly improved spatial learning and reduced IL-6 (p < 0.05), whereas *Glycyrrhiza glabra* had limited effects. The combination therapy yielded the strongest outcomes, significantly reducing escape latency (p < 0.01), IL-6 and TNF-α levels (p < 0.01 and p < 0.05), and downregulating APP and MAPT expression. Additionally, oxidative damage was attenuated, as indicated by decreased MDA and elevated SOD activity (p < 0.001). Although direct measurement of TLR4/NF-κB phosphorylation was not performed, the observed anti-inflammatory and antioxidant effects suggest possible modulation of this pathway.

**Conclusion:**

Co-administration of curcumin and *Glycyrrhiza glabra* exerts synergistic neuroprotective effects by attenuating neuroinflammation, oxidative stress, and AD-related protein expression, surpassing monotherapy outcomes. These findings suggest a synergistic neuroprotective mechanism via modulation of TLR4/MyD88/NF-κB and redox pathways. This study provides preclinical evidence supporting the development of TCM-based combination strategies for AD intervention.

## 1 Introduction

Alzheimer’s disease (AD) is a progressive neurodegenerative disorder primarily characterized by cognitive decline, memory impairment, and behavioral abnormalities. Its neuropathological hallmarks include extracellular β-amyloid (Aβ) plaques and intracellular neurofibrillary tangles (NFTs) composed of hyperphosphorylated tau protein, leading to synaptic degeneration and neuronal dysfunction (Graff-Radford et al., 2021). With the global population aging rapidly, the prevalence of AD is increasing, posing significant medical and socioeconomic challenges (2023). Although current pharmacotherapies such as acetylcholinesterase inhibitors and NMDA receptor Antagonists offer temporary symptomatic relief, they fail to modify the disease course or halt neurodegeneration (Khan et al., 2020; Yiannopoulou and Papageorgiou, 2020; Congdon et al., 2023; Tahami Monfared et al., 2023). This has prompted the search for novel, safe, and multi-targeted therapeutic strategies. TCM emphasizes holistic regulation and multi-component synergy, offering a potential advantage in the management of complex diseases such as AD. Among TCM-derived agents, curcumin (a major constituent of *Curcuma longa*) and glycyrrhizic acid (from *Glycyrrhiza glabra*) have attracted interest due to their reported anti-inflammatory and antioxidant activities (Azzini et al., 2024; Song et al., 2025). Curcumin has been shown to inhibit Aβ aggregation, modulate tau phosphorylation, and attenuate neuroinflammation by regulating NF-κB and MAPK signaling cascades (Baghcheghi et al., 2024; Piekarz et al., 2024; Sen et al., 2024). Glycyrrhiza glabra, on the other hand, exhibits immune-modulating and anti-inflammatory properties, though its role in AD pathogenesis remains less defined (Eltahir et al., 2024; Fan et al., 2025). In TCM pharmacology, curcumin and *Glycyrrhiza glabra* are frequently combined based on the Jun–Zuo principle, wherein *C. longa* acts as the principal herb to disperse blood stasis and reduce inflammation, while *Glycyrrhiza glabra* serves as the assistant herb to potentiate efficacy and minimize toxicity. Recent studies suggest that this pairing may exert synergistic effects by targeting complementary nodes within inflammatory signaling pathways, particularly Toll-like receptor 4 (TLR4) and nuclear factor-kappa B (NF-κB) (Chen et al., 2021; Guo et al., 2022). Curcumin inhibits NF-κB activation by stabilizing IκBα, whereas glycyrrhizic acid modulates upstream TLR4 signaling via suppression of damage-associated molecular patterns such as HMGB1 (Verma et al., 2024). Despite accumulating evidence of their individual efficacy, the synergistic interaction between curcumin and glycyrrhizic acid in AD remains inadequately characterized. Moreover, variability in formulation, bioavailability, and intervention timing complicates cross-study interpretation (Chatterjee et al., 2018). Therefore, this study aims to systematically investigate the neuroprotective effects of curcumin and *Glycyrrhiza glabra*, individually and in combination, in a D-galactose/sodium nitrite-induced mouse model ofAD. We assess their effects on cognitive function, neuroinflammation, oxidative stress, and the expression of AD-related proteins (APP and MAPT), with a focus on potential modulation of the TLR4/MyD88/NF-κB and redox pathways. These findings are expected to provide preclinical insight into the rational design of TCM-based combinatorial interventions for AD.

## 2 Materials and methods

### 2.1 Animal experiments

#### 2.1.1 Animal source and housing conditions

Fifty specific pathogen-free (SPF) C57BL/6 mice (25 males and 25 females, aged 6–8 weeks) were purchased from Hunan Slack Jingda (Hunan, China). All mice were housed under standard laboratory conditions (22 °C–26 °C, 50%–60% relative humidity, 12-h light/dark cycle) with *ad libitum* access to food and water. Animals were randomly assigned to five experimental groups (n = 10 per group; five males and five females), ensuring sex balance to reduce gender-related variability in behavioral and inflammatory outcomes. Group size was determined based on power analysis from previous behavioral and molecular studies (power ≥0.8), while complying with ethical and feasibility constraints. *The animal study was approved by Wuhan Myhalic Biotechnological Co., Ltd. for Nationalities Institutional Review Board (SYXK 2018-0104). The study was conducted in accordance with the local legislation and institutional requirements.*

#### 2.1.2 Grouping and model induction

Mice were randomly divided into the following five groups.

- wild-type (WT) Group (Control Group): Intraperitoneal injection of an equal volume of normal saline. This group served as the baseline physiological control without exposure to modeling agents or treatments.
- AD Model Group: Daily intraperitoneal injection of D-galactose (120 mg/kg) and sodium nitrite (45 mg/kg).
- AD + Curcumin Group: Received D-galactose and sodium nitrite as above, plus oral curcumin (100 mg/kg).
- AD + Glycyrrhiza *glabra* Group: Received D-galactose and sodium nitrite, plus oral Glycyrrhiza glabra extract (100 mg/kg).
- AD + Combination Group: Received D-galactose and sodium nitrite, plus oral curcumin (100 mg/kg) and Glycyrrhiza glabra extract (100 mg/kg).

All interventions were administered once daily for 8 weeks.

#### 2.1.3 Sample collection

At the end of the treatment period, mice were anesthetized with 1% sodium pentobarbital. Upon confirmation of complete cessation of cardiac and respiratory function, blood samples and brain tissues were collected for subsequent analysis. All procedures were performed by certified laboratory personnel in accordance with the ethical standards approved by the Animal Management Committee of Hubei Province.

### 2.2 Morris water maze (MWM) test

The Morris water maze test consisted of two parts: the hidden platform test (spatial acquisition phase) and the probe trial (spatial exploration phase).

- Hidden Platform Test (Spatial Acquisition Phase)

Prior to the test, mice from all five groups were placed on the platform in the third quadrant for a 20-s adaptation period. Subsequently, each mouse was randomly placed into the pool, facing the wall, from different quadrants to begin the timed trial.

The trial was completed once the mouse successfully reached the platform and remained on it for 5 s. The maximum trial duration was set at 60 s. If a mouse failed to locate the platform within 60 s, it was manually guided to the platform and allowed to adapt for 10 s. After each trial, the mice were dried and returned to their cages. The test was conducted over seven consecutive days.

- Probe Trial (Spatial Exploration Phase)

On the fifth day of spatial acquisition training, the probe trial was conducted while maintaining the same environmental conditions and water temperature. The submerged platform in the third quadrant was removed, and the mice were placed into the pool from the first quadrant, facing the wall. A computerized tracking system recorded and analyzed the swimming trajectory of each mouse over a 60-s period. The number of times the mouse crossed the former platform location and the percentage of time spent in the third quadrant (target quadrant) were measured to assess spatial memory ability.

### 2.3 Western blot analysis of protein expression in mice

Brain tissues were homogenized in lysis buffer containing protease and phosphatase inhibitors. After protein extraction and quantification using a bicinchoninic acid (BCA) assay kit (Beyotime), equal amounts of protein (30 μg per sample) were separated via SDS-PAGE and transferred onto PVDF membranes. Membranes were blocked with 5% non-fat milk and incubated overnight at 4 °C with primary antibodies against IL-6, TNF-α, APP, or MAPT (Bioswamp). After washing, membranes were incubated with HRP-conjugated secondary antibodies (1:5000) for 2 h at room temperature. Protein bands were visualized using chemiluminescent substrate, and images were captured for densitometric analysis.

### 2.4 Real-time PCR analysis of RNA expression in mice

Total RNA was extracted from 100 mg of mouse brain tissue using RNAiso Plus (TAKARA). RNA purity and concentration were assessed spectrophotometrically. cDNA synthesis was performed using the PrimeScript™ RT reagent kit (TAKARA) with 600 ng total RNA input. qPCR was conducted using SYBR Green Master Mix (YEASEN) with the following gene-specific primers.

**Table:** 

Gene	Forward primer (5′–3′)	Reverse primer (5′–3′)
IL-6	CCT​ACC​CCA​ATT​TCC​AAT​GCT​C	GGT​CTT​GGT​CCT​TAG​CCA​CTC
TNF-α	GGT​GCC​TAT​GTC​TCA​GCC​TCT​T	GCC​ATA​GAA​CTG​ATG​AGA​GGG​AG
MAPT	CCT​GAG​CAA​AGT​GAC​CTC​CAA​G	CAA​GGA​GCC​AAT​CTT​CGA​CTG​G
APP	TGC​AGC​AGA​ACG​GAT​ATG​AG	ACA​CCG​ATG​GGT​AGT​GAA​GC
Tau	CCT​GAG​CAA​AGT​GAC​CTC​CAA​G	CAA​GGA​GCC​AAT​CTT​CGA​CTG​G
GAPDH (ref.)	GTG​TTC​CTA​CCC​CCA​ATG​TGT	ATT​GTC​ATA​CCA​GGA​AAT​GAG​CTT

Gene expression was normalized to GAPDH, and analyzed using the 2^−ΔΔCt^, method.

### 2.5 Lipid peroxidation (MDA) and total superoxide dismutase (sod) activity assay

Venous blood was collected via tail vein and centrifuged to obtain plasma. Lipid peroxidation was assessed by measuring malondialdehyde (MDA) using a colorimetric assay kit (Bioswamp). MDA content was determined based on absorbance at 532 nm, compared to a standard curve.

Total Superoxide Dismutase(SOD)activity was measured using a WST-based assay kit (Bioswamp). After dilution, the reaction mixture was incubated at 37 °C for 30 min, and absorbance was measured at 450 nm. SOD activity was expressed in units per milligram of total protein.

### 2.6 Major reagents and chemicals

Key reagents included.

- D-(+)-Galactose, sodium nitrite (Shanghai Aladdin)
- Curcumin and Glycyrrhiza glabra extracts (Xi’an YunYue Biotechnology)
- MDA and SOD Assay Kits (Bioswamp)
- RNAiso Plus, PrimeScript RT Kit (TAKARA)
- qPCR Master Mix (YEASEN)
- Antibodies (Bioswamp): IL-6, TNF-α, MAPT, APP
- Protein marker, BCA kit, and lysis buffer (Thermo Fisher and Beyotime)

### 2.7 Statistical analysis

All statistical analyses were performed using SPSS v27.0 and GraphPad Prism v6.0.

Data were presented as mean ± tandard deviation (SD). Group comparisons were analyzed using one-way analysis of variance (ANOVA) followed by Tukey’s *post hoc* test. Two-way ANOVA was applied to assess interaction effects. For nonparametric data (e.g., quadrant crossings), Kruskal–Wallis test was used (H = 36.48, p = 2.30 × 10^−7^).

Escape latency was capped at 60 s per trial in accordance with standard MWM protocols. Individual data distributions were visualized using box-and-whisker plots.

All tests were two-tailed, and significance was set at p < 0.05. Effect sizes and exact p-values were reported to enhance interpretability and reproducibility.

## 3 Results

### 3.1 Behavioral analysis

The MWM test was employed to assess spatial learning and memory function in mice. As shown in Table 1, mice in the AD model group displayed a significantly prolonged escape latency (p < 0.01) and a reduced number of entries into the target (third) quadrant (p < 0.01) compared to the WT control group, indicating impaired spatial cognition.

**TABLE 1:** Comparison of morris water maze behavioral performance among groups.

Group	Escape latency (s)	Average swimming speed (mm/s)	Number of entries into the third quadrant
WT (Control)	22.89 ± 3.39**	217.61 ± 2.54**	3.8 ± 0.75**
AD Model	60	132.11 ± 27.0	1.40 ± 0.49
AD + CL	60	148.71 ± 11.23	1.83 ± 0.4
AD + GG	60	105.67 ± 13.09	2.33 ± 0.8
AD + Combination	41.54 ± 8.37**	204.71 ± 24.83**	4.20 ± 1.32*

Compared with the AD, group *:p < 0.05; **:p < 0.01; *Glycyrrhiza glabra (GG): Curcuma longa (CL)*.

Curcumin monotherapy moderately improved escape latency (p < 0.05) but did not significantly enhance spatial exploration. Glycyrrhiza glabra treatment alone had a marginal effect on swimming speed and quadrant entries, though not statistically significant. Notably, the combination therapy group (Curcuma longa + Glycyrrhizaglabra) exhibited the most pronounced behavioral improvements, with a significantly shorter escape latency (p < 0.01), near-normalized swimming speed (p < 0.01), and a significantly increased number of entries into the target quadrant (p < 0.05) compared to the AD model group.

These results suggest that the co-administration of curcumin and Glycyrrhiza glabra exerts synergistic effects in restoring spatial memory and learning in AD mice.

The Morris water maze was used to evaluate spatial learning and memory. Compared with wild-type (WT) mice, the AD model group displayed significantly prolonged escape latencies and reduced target quadrant entries (p < 0.01), indicating cognitive impairment. Curcumin (CL) treatment moderately improved escape latency (p < 0.05), whereas Glycyrrhiza glabra (GG) alone had minimal impact. Notably, the CL + GG combination produced the most pronounced improvements, with escape latency markedly shortened (p < 0.01) and target quadrant crossings significantly increased (p < 0.05) compared with the AD group.

**FIGURE 1:**
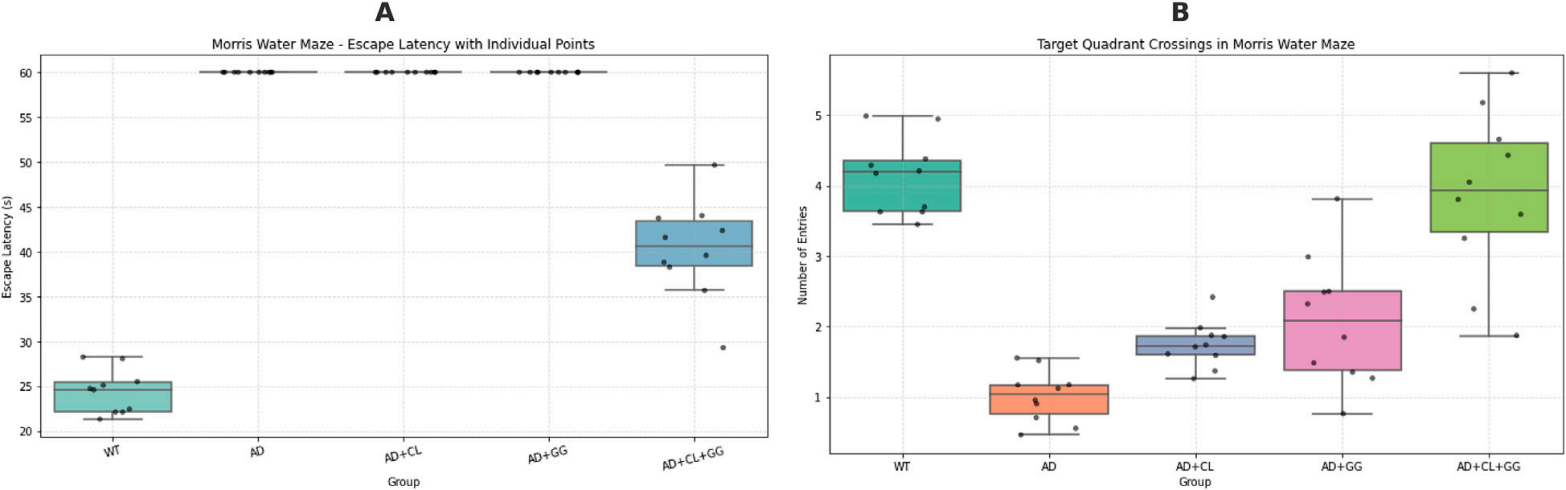
Behavioral performance in the Morris water maze: escape latency and targetquadrant crossings across treatment groups. **(A)** Escape latency (s) during the acquisition phase. Data are presented as box-and-whisker plots (median and interquartile range) with individual values (n = 10 per group). Trials in which mice failed to locate the hidden platform within 60 s were assigned the maximum latency according to standard protocols. The AD model group showed markedly prolonged escape latencies compared with wild-type (WT) mice (p < 0.01), indicating impaired spatial learning. Treatment with curcumin (CL), Glycyrrhiza glabra (GG), and particularly the combined CL + GG intervention significantly shortened escape latencies, reflecting improved learning performance. **(B)** Target quadrant crossings during the probe trial. Data are presented as boxplots with individual data points. To account for potential right-censoring bias in escape latency, both parametric and non-parametric analyses were performed. Kruskal–Wallis testing revealed a highly significant group effect (H = 36.48, p = 2.30 × 10^−7^), confirming that intergroup variability exceeded assumptions of normality. AD model mice exhibited significantly fewer target quadrant entries compared with WT (p < 0.01), whereas treatment groups showed improvements, with the CL + GG combination group displaying the strongest effect (p < 0.05 vs. AD). *Statistical analysis: one-way ANOVA and Kruskal–Wallis test. *p < 0.05, *p < 0.01 vs. *WT; #p < 0.05, ##p < 0.01* vs. *AD.*

To account for potential bias due to latency capping at 60 s, additional non-parametric analysis was conducted. The Kruskal–Wallis test confirmed a highly significant group effect (H = 36.48, p = 2.30 × 10^−7^), supporting the robustness of the observed intergroup differences. These findings demonstrate that co-administration of curcumin and Glycyrrhiza glabra exerts synergistic benefits on spatial learning and memory performance (Figure 1).

### 3.2 Expression of inflammatory cytokines

Quantitative PCR analysis revealed that mice in the AD model group exhibited significantly elevated mRNA expression levels of pro-inflammatory cytokines IL-6 and TNF-α compared to the WT control group (p < 0.01), confirming the induction of a neuroinflammatory state.

Curcumin monotherapy significantly downregulated IL-6 expression (p < 0.05), but had no statistically significant effect on TNF-α levels (p > 0.05), suggesting a partial anti-inflammatory effect. Glycyrrhiza glabra monotherapy showed no significant modulation of either cytokine.

In contrast, the combination therapy (Curcuma longa + Glycyrrhiza glabra) produced a more robust anti-inflammatory response, with significant reductions observed in both IL-6 (p < 0.001) and TNF-α (p < 0.01) levels. These findings indicate a synergistic interaction between curcumin and glycyrrhizic acid, resulting in enhanced suppression of neuroinflammatory mediators (Table 2).

**TABLE 2:** Relative mRNA expression of IL-6, TNF-α, app, and MAPT (normalized to AD group = 1).

Group	IL-6	TNF-α	App	MAPT
WT (Control)	0.15 ± 0.01***	0.22 ± 0.02***	0.36 ± 0.02	0.38 ± 0.00***
AD Model	1	1	1	1
AD + CL	0.63 ± 0.04***	1.40 ± 0.19**	0.82 ± 0.05	0.93 ± 0.08**
AD + GG	0.93 ± 0.04	1.05 ± 0.03	1.02 ± 0.10	1.04 ± 0.20
AD + Combination	0.30 ± 0.01***	0.36 ± 0.02***	0.43 ± 0.05***	0.34 ± 0.01***

Compared with the AD group *:p < 0.05; **:p < 0.01; ***:p < 0.001 (n = 10, $\bar{x}$ ± s); *Glycyrrhiza glabra (GG): Curcuma longa (CL)*.

### 3.3 Expression of AD-related proteins

**FIGURE 2:**
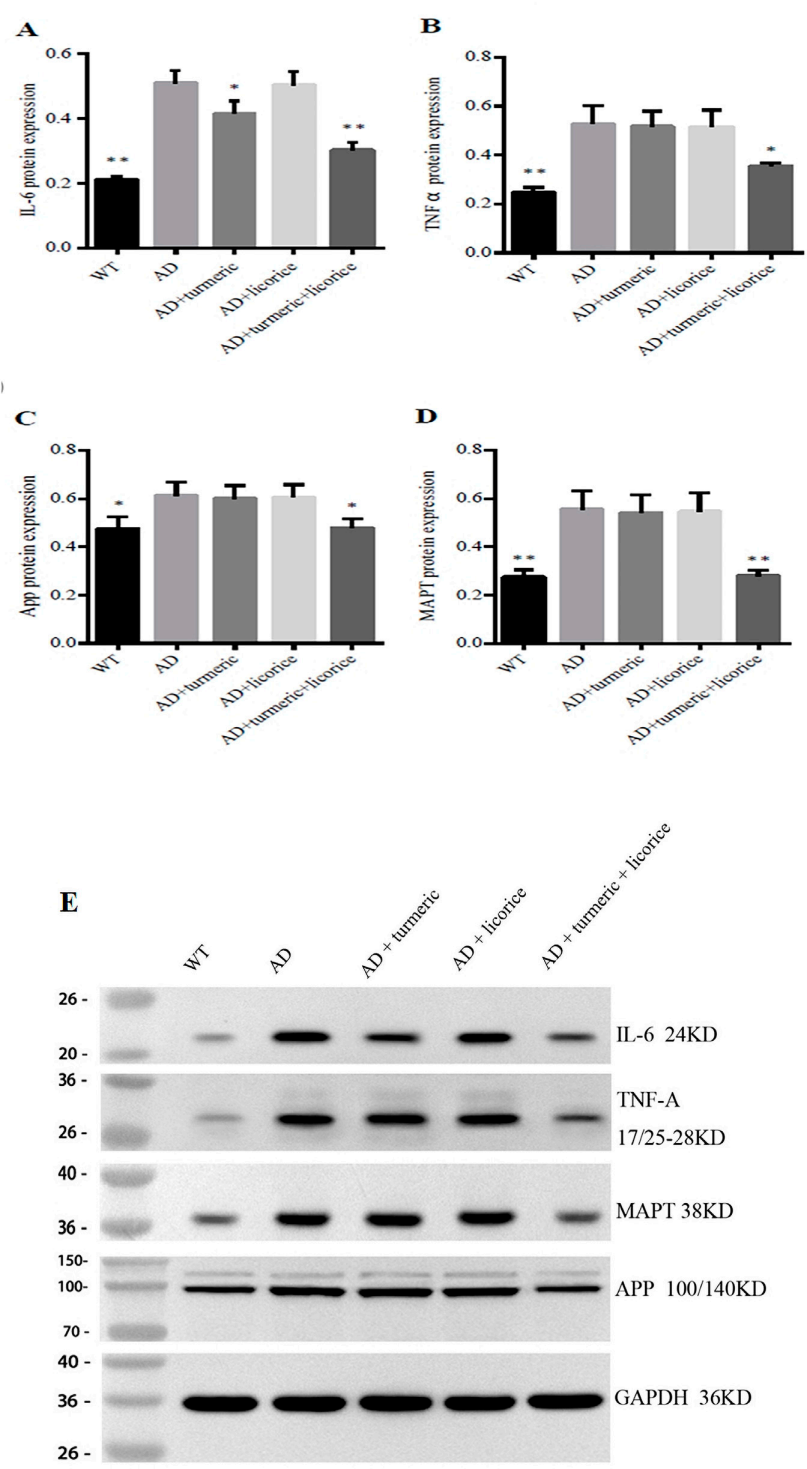
Turmeric and *Glycyrrhiza glabra* Modulate Brain Inflammation and AD Proteins in Mice. Western blot analysis of hippocampal proteins in each group **(A)** IL-6 **(B)** TNF-α **(C)** APP, and **(D)** MAPT protein levels, normalized to GAPDH, are shown as quantitative bar graphs. **(E)** Representative Western blot bands for IL-6 (24 kDa), TNF-α (17/25–28 kDa), MAPT (38 kDa), and APP (100/140 kDa), with GAPDH (36 kDa) as the loading control. Data are presented as mean ± SD (n = 10 per group). Compared with wild-type (WT) controls, the AD model group exhibited significantly elevated IL-6, TNF-α, APP, and MAPT levels. Monotherapy with curcumin (CL) or Glycyrrhiza glabra (GG) produced only modest or non-significant changes. By contrast, the combined CL + GG treatment markedly suppressed both inflammatory cytokines and AD-related proteins, consistent with a synergistic therapeutic effect. *Statistical analysis: Student’s t-test. *p < 0.05, **p < 0.01, **p < 0.001 versus AD group.

Western blot analysis demonstrated that the AD model group exhibited markedly elevated protein levels of APP and MAPT compared with wild-type controls (p < 0.01), confirming the establishment of AD-like pathology (Table 3; Figure 2). Treatment with curcumin alone produced only slight, non-significant reductions in APP and MAPT expression (p > 0.05), indicating limited efficacy of monotherapy in mitigating AD-related protein burden. Glycyrrhiza glabra monotherapy similarly failed to produce statistically significant suppression, though a minor downward trend was observed.

**TABLE 3:** Relative Protein Expression of IL-6, TNF-α, APP, and MAPT in Mouse Brain Tissue (Western blot, normalized to AD group = 1).

Group	IL-6	TNF- α	App	MAPT
WT (Control)	0.21 ± 0.007**	0.25 ± 0.02**	0.47 ± 0.04*	0.28 ± 0.02**
AD Model	0.51 ± 0.03	0.53 ± 0.06	0.61 ± 0.05	0.56 ± 0.06
AD + CL	0.42 ± 0.03*	0.52 ± 0.06	0.60 ± 0.04	0.54 ± 0.06
AD + GG	0.50 ± 0.03	0.52 ± 0.06	0.61 ± 0.04	0.54 ± 0.06
AD + Combination	0.30 ± 0.02**	0.35 ± 0.02*	0.48 ± 0.03*	0.28 ± 0.02**

Compared with the AD group *:p < 0.05; **:p < 0.01; ***:p < 0.001 (n = 10, $\bar{x}$ ±s); *Glycyrrhiza glabra (GG): Curcuma longa (CL)*.

In contrast, the combination therapy resulted in significant attenuation of both APP (p < 0.05) and MAPT (p < 0.01), suggesting a synergistic effect in reducing amyloidogenic and tau-related pathology. In addition, consistent with earlier mRNA findings, AD model mice displayed elevated IL-6 and TNF-α protein levels relative to controls, further validating the inflammatory and neurodegenerative phenotype. Importantly, co-administration of curcumin and Glycyrrhiza glabra significantly suppressed IL-6 (p < 0.01) and TNF-α (p < 0.05) compared with the AD group, whereas monotherapies showed only modest or non-significant effects. Collectively, these results highlight that curcumin and Glycyrrhiza glabra act synergistically to suppress neuroinflammation and reduce AD-related protein accumulation, with the combination therapy demonstrating superior efficacy compared with either agent alone.

### 3.4 Combined administration of curcumin and *Glycyrrhiza glabra* mitigates aging and enhances antioxidant capacity in AD mice

To evaluate antioxidant defense and oxidative damage, systemic SOD activity and MDA levels were measured. Compared with WT controls, AD model mice exhibited significantly reduced SOD activity and elevated MDA levels (p < 0.01), confirming impaired antioxidant capacity and enhanced oxidative stress.

Treatment with CL or GG alone partially restored antioxidant status, as reflected by increased SOD activity and decreased MDA levels (p < 0.01 vs. AD). Notably, the combined CL + GG intervention exerted the most pronounced effect, producing a highly significant elevation in SOD activity and a marked reduction in MDA concentration (p < 0.001 vs. AD).

**FIGURE 3:**
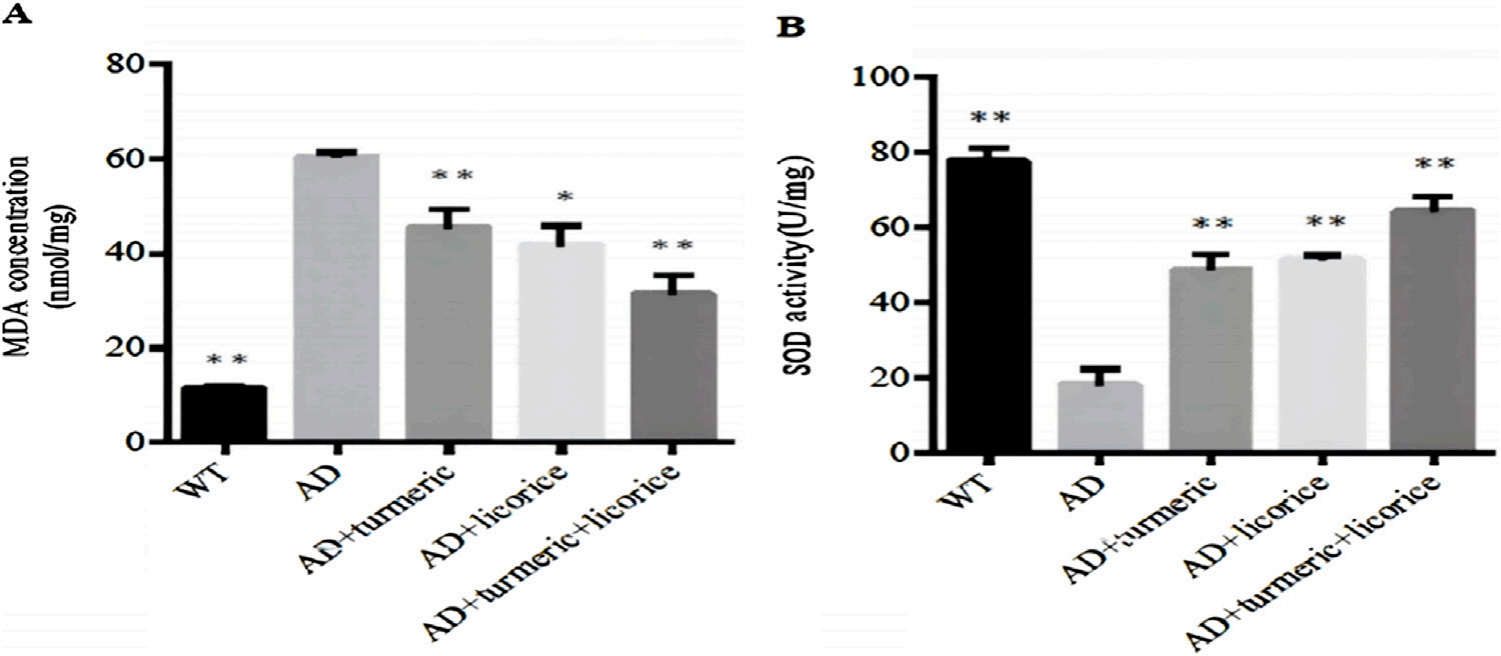
Expression levels of MDA and SOD across experimental groups. **(A)** MDA concentration and **(B)** SOD activity in serum were measured to assess systemic lipid peroxidation and antioxidant capacity. Data are presented as mean ± SD (n = 10 per group). Compared with wild-type (WT) controls, AD model mice exhibited elevated MDA levels and reduced SOD activity. Treatment with curcumin (CL) or Glycyrrhiza glabra (GG) alone partially restored antioxidant status, while the combined CL + GG therapy produced the strongest effect, with a significant decrease in MDA and a robust increase in SOD activity. *Statistical analysis: one-way ANOVA. *p < 0.05, *p < 0.01 versus AD group.

These results indicate that co-administration of curcumin and Glycyrrhiza glabra provides enhanced protection against oxidative stress and aging-associated biochemical changes in AD mice (Figure 3).

### 3.5 Integrated analysis of inflammatory cytokines and AD-related protein expression

Curcumin monotherapy exerted a modest inhibitory effect on IL-6 expression (*p* < 0.05), while showing no significant impact on TNF-α, APP, or MAPT expression, indicating limited anti-inflammatory and anti-amyloid effects when used alone. Similarly, *Glycyrrhiza glabra* monotherapy failed to produce statistically significant changes across all measured biomarkers, suggesting that its standalone efficacy in modulating AD-related neuroinflammation and protein aggregation is minimal. In contrast, the combination treatment with curcumin and *Glycyrrhiza glabra* elicited the most robust therapeutic effects. Levels of both IL-6 and TNF-α were significantly reduced (*p* < 0.01and *p* < 0.05, respectively), indicative of enhanced anti-inflammatory activity. Moreover, the expression of APP and MAPT—key proteins implicated in Aβ deposition and tau hyperphosphorylation—was significantly downregulated (*p* < 0.05 and *p* < 0.01, respectively), suggesting that the co-treatment may alleviate AD-related pathological protein accumulation (Table 4).

**TABLE 4:** Summary of therapeutic effects on inflammatory and AD-Related biomarkers.

Comparison	IL-6	TNF− α	App	MAPT
AD VS WT	↑ ↑	↑	↑	↑ ↑
AD VS CL	↓(p < 0.05)	_→_	_→_	↓(p > 0.05)
AD VS GG	_→_	_→_	_→_	_→_
AD VS Combination	↓ ↓(p < 0.01)	↓(p < 0.05)	↓ (p < 0.05)	↓ ↓(p < 0.01)

↑, Increased; ↑↑, Significantly increased; →, no change; ↓, decreased; ↓↓, significantly decreased; GG, *Glycyrrhiza glabra; CL, Curcuma longa*.

### 3.6 Significant interaction between curcumin and *Glycyrrhiza glabra*

Two-way ANOVA revealed that both CL and GG monotherapies exerted significant main effects on the expression of IL-6, TNF-α, APP, and MAPT (all p < 0.01). More importantly, a highly significant interaction effect was detected between CL and GG across all measured biomarkers (interaction p < 0.0001), indicating that their combined action is synergistic rather than additive.

These synergistic effects were reflected by greater reductions in pro-inflammatory cytokines (IL-6, TNF-α) and AD-related proteins (APP, MAPT) in the CL + GG group compared with either monotherapy. The interaction patterns support the hypothesis that co-administration enhances therapeutic efficacy by targeting overlapping molecular mechanisms.

**FIGURE 4:**
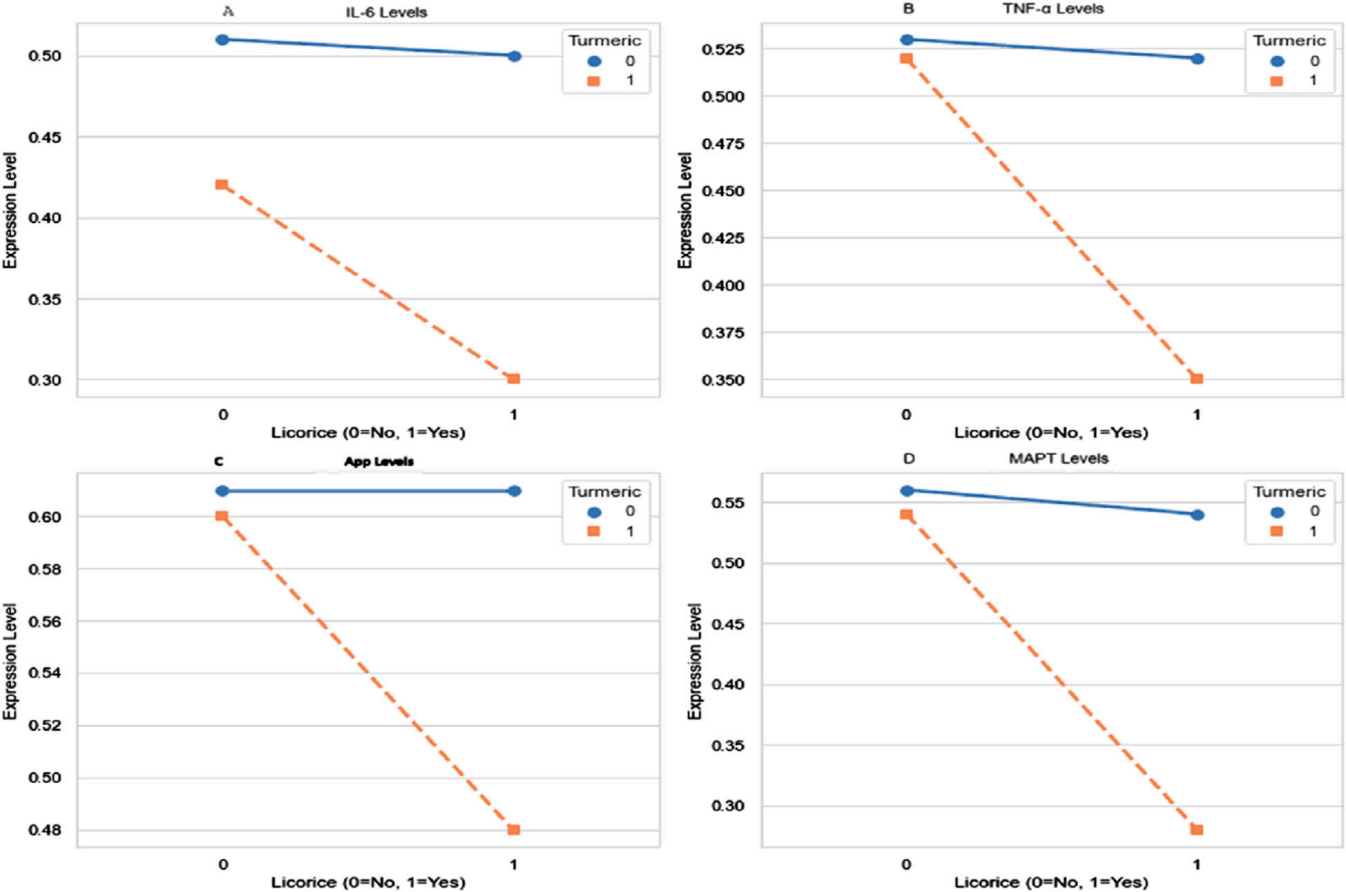
Interaction effects of curcumin and glycyrrhiza *glabra* on IL-6, TNF-α, APP, MAPT. Two-way ANOVA was performed to evaluate main and interaction effects of curcumin (CL) and Glycyrrhiza glabra (GG) on protein expression. Significant main effects of both agents were observed for **(A)** IL-6 **(B)** TNF-α **(C)** APP, and **(D)** MAPT levels. Notably, highly significant interaction effects (p < 0.0001) were detected across all biomarkers, indicating that the combined CL + GG intervention produced synergistic rather than merely additive effects. Data are expressed as mean values Statistical analysis: two-way ANOVA. Main effects and interaction effects were all significant at p < 0.01; interaction p-values <0.0001.

Collectively, these findings suggest potential crosstalk between curcumin- and glycyrrhizic acid-mediated pathways, possibly converging on TLR4/MyD88/NF-κB signaling and tau phosphorylation cascades (Figure 4).

## 4 Discussion

AD is a progressive neurodegenerative disorder primarily characterized by memory loss, cognitive decline, and neuropathological features such as Aβ accumulation and tau hyperphosphorylation. While current clinical therapies provide symptomatic relief, they do not alter disease progression. In recent years, TCM has garnered increasing attention for its multi-targeted and synergistic therapeutic properties in complex disorders like AD.

This study evaluated the individual and combined neuroprotective effects of curcumin and *Glycyrrhiza glabra* in a D-galactose/sodium nitrite-induced mouse model of AD. Curcumin monotherapy yielded modest benefits in spatial learning and selectively attenuated IL-6 expression. In contrast, *Glycyrrhiza glabra* alone showed negligible therapeutic effect. Notably, combination treatment significantly improved cognitive performance, reduced neuroinflammatory cytokine levels (IL-6 and TNF-α), decreased expression of AD-related proteins (APP and MAPT), and restored redox balance, as evidenced by increased SOD activity and reduced MDA content. These results suggest that curcumin and *Glycyrrhiza glabra* exert synergistic neuroprotection in AD, likely through multi-targeted modulation of inflammatory, oxidative, and other pathological cascades.

Cognitive impairment is the most prominent clinical manifestation of AD. In this study, the MWM test was employed to evaluate cognitive function in AD model mice. The results showed that escape latency was significantly prolonged and time spent in the target quadrant was markedly reduced in the AD group (*p* < 0.01), indicating that AD-related pathological changes had impaired spatial learning and memory function (Chatterjee et al., 2018). Curcumin monotherapy significantly shortened escape latency (*p* < 0.05),but did not produce a notable improvement in spatial exploration ability. Treatment with licorice alone had no significant effect. In contrast, combined administration of curcumin and licorice led to a marked reduction in escape latency (*p* < 0.01) and significantly enhanced spatial exploration performance (*p* < 0.05), demonstrating that the combination therapy was more effective in improving cognitive function than either treatment alone.

To further explore the molecular mechanisms underlying the observed improvements in cognition and biomarker profiles, we focused on known inflammatory and oxidative stress pathways implicated in AD pathogenesis. Curcumin (Curcuma longa) has demonstrated robust neuroprotective effects, including attenuation of Aβ accumulation, inhibition of tau phosphorylation, and enhancement of synaptic plasticity (Sabouni et al., 2023; Perales-Salinas et al., 2024). In contrast, however, its monotherapy efficacy in AD models appears limited. Consistent with the TCM pairing principle of “Jun-Zuo” (Zeng et al., 2024). our findings suggest that Glycyrrhiza glabra may potentiate curcumin’s actions via mechanistically distinct yet synergistic pathways.

One of the key mechanisms underlying AD progression is neuroinflammation. The TLR4/NF-κB signaling pathway orchestrates microglial activation and regulates the transcription of pro-inflammatory mediators such as IL-6 and TNF-α (Liu et al., 2022; Udeochu et al., 2023; Wang et al., 2024). In our model, curcumin alone significantly reduced IL-6 levels (p < 0.05) without affecting TNF-α, while Glycyrrhiza glabra monotherapy showed negligible impact. However, combination therapy markedly suppressed both cytokines, indicating a broader anti-inflammatory effect.

Mechanistically, curcumin suppresses NF-κB signaling by stabilizing IκBα, thereby preventing the nuclear translocation of the p65 subunit (Esmaealzadeh et al., 2024). Meanwhile, glycyrrhizic acid downregulates upstream DAMPs—particularly HMGB1—thereby attenuating TLR4 activation (Wu et al., 2022; Li et al., 2024; Lou et al., 2025). These actions converge at the adaptor protein MyD88, which recruits IRAK4/TRAF6 to propagate the inflammatory cascade (Cai et al., 2021; Maurya et al., 2025). Thus, this dual-site inhibition—upstream at TLR4 and downstream at NF-κB—may explain the synergistic efficacy of the combination therapy. However, as phosphorylation and nuclear translocation of NF-κB components were not directly assessed, this model remains hypothetical and warrants further molecular validation (Zhang et al., 2021; Sivamaruthi et al., 2023).

Beyond inflammation, oxidative stress is another central pathological factor in Alzheimer’s disease. To further investigate the antioxidant potential of curcumin and *Glycyrrhiza glabra*, we measured brain levels of MDA, an indicator of lipid peroxidation, and SOD, a key antioxidant enzyme. Our results showed that AD model mice exhibited significantly increased MDA and decreased SOD activity compared to controls, indicating elevated oxidative stress. Monotherapy with either curcumin or *Glycyrrhiza glabra* partially reversed these changes (p < 0.01), while the combination treatment produced the most pronounced improvement (p < 0.001), suggesting a synergistic antioxidative effect. These findings further support the neuroprotective potential of curcumin–licorice co-administration through modulation of redox homeostasis.

However, we acknowledge that MDA and SOD alone, while classical and informative, represent only a subset of the oxidative stress spectrum. A more comprehensive redox profile would benefit from additional biomarkers. For example, the glutathione redox ratio (GSH/GSSG) provides a sensitive measure of cellular oxidative imbalance and mitochondrial dysfunction, while CAT and glutathione peroxidase (GPx) reflect enzymatic detoxification of hydrogen peroxide and other reactive oxygen species. In future work, we plan to include these markers in both cortical and hippocampal tissues to better characterize redox dysregulation during AD progression, and to assess how combinatorial herbal therapy modulates intracellular oxidative networks. Such a multi-marker approach may enhance mechanistic interpretation and clarify the interplay between oxidative injury, neuroinflammation, and neurodegeneration.

The abnormal accumulation of Aβ plaques and tau tangles is a pathological hallmark of AD, contributing to synaptic dysfunction and neuronal loss through chronic inflammation and microtubule destabilization (Scheltens et al., 2021; Ossenkoppele et al., 2022). In our study, curcumin monotherapy had a limited effect on APP and MAPT expression, while the combination treatment significantly downregulated both proteins (p < 0.05, p < 0.01, respectively), suggesting dual interference with Aβ accumulation and tau hyperphosphorylation. Mechanistically, curcumin is known to inhibit BACE1 and modulate tau via CDK5/GSK-3β, whereas glycyrrhizic acid may further regulate tau through the Akt/GSK-3β signaling pathway (Zheng et al., 2017). These convergent pathways may underlie the observed synergistic suppression of AD-related protein expression.

This study offers preliminary insights into the neuroprotective mechanisms of curcumin and *Glycyrrhiza glabra* co-administration in a D-galactose/sodium nitrite-induced mouse model of Alzheimer’s disease. While our findings support their synergistic efficacy, several limitations merit discussion. First, the involvement of the TLR4/NF-κB pathway remains hypothetical, as we did not directly assess p65 phosphorylation, IκBα degradation, or nuclear translocation. Future studies should include molecular assays such as Western blotting and immunofluorescence to validate this mechanism. Second, behavioral assessment was limited to a single endpoint in the Morris Water Maze. A repeated-measures design would allow finer tracking of learning progression. Third, we did not characterize the pharmacokinetics of either compound, despite known issues with poor oral bioavailability and limited brain access. Delivery optimization strategies (e.g., nanoparticles or piperine-enhanced formulations) are needed. Fourth, only a single dose (100 mg/kg) was tested in this study. Although selected based on prior literature, the absence of a dose–response analysis limits the understanding of the therapeutic window and optimal dosing parameters. A graded dosing scheme is recommended to define efficacy thresholds and assess potential toxicity. Fifth, this study focused exclusively on short-term therapeutic outcomes. Longitudinal studies with extended follow-up periods are necessary to determine the durability and long-term safety of the observed benefits. Sixth, molecular-level findings were not corroborated by histopathological data. Future studies should incorporate tissue-based techniques such as hematoxylin–eosin (H&E) staining, immunohistochemistry (IHC), or immunofluorescence (IF) to localize protein expression and visualize neuroinflammatory and neurodegenerative changes. Finally, the D-galactose/sodium nitrite-induced model used in this study partially replicates features of aging-related oxidative stress but does not fully capture the complexity of human AD pathology, particularly Aβ plaque and tau tangle formation.

Future validation in transgenic AD models (e.g., APP/PS1, 3xTg-AD) is warranted. Moreover, expanding investigations to other neurodegenerative conditions such as Parkinson’s disease or frontotemporal dementia may enhance the translational relevance of the combined therapeutic strategy.

## 5 Conclusion

This study demonstrates that the combined administration of curcumin and *Glycyrrhiza glabra* yields superior therapeutic outcomes compared to either monotherapy in a D-galactose/sodium nitrite-induced mouse model of AD. The co-treatment significantly enhanced spatial learning and memory, attenuated neuroinflammatory cytokines (IL-6 and TNF-α), downregulated AD-associated proteins (APP and MAPT), and restored antioxidant capacity by increasing SOD activity and decreasing MDA levels.

These neuroprotective effects appear to arise from the synergistic modulation of key pathological cascades, including the TLR4/MyD88/NF-κB signaling pathway and redox imbalance. Additionally, the combination therapy may concurrently target both amyloidogenic (via BACE1 inhibition) and tau-related (via GSK-3β/CDK5 modulation) mechanisms, offering broader efficacy against hallmark features of AD pathology. However, further molecular validation is required to confirm these mechanistic pathways. Collectively, these findings support the hypothesis that multi-targeted co-treatment strategies derived from TCM may offer a promising approach for the prevention or treatment of AD. Future research should focus on mechanistic elucidation, pharmacokinetic optimization, and validation in transgenic AD models to facilitate clinical translation.

## Data Availability

The datasets presented in this study can be found in online repositories. The names of the repository/repositories and accession number(s) can be found below: https://figshare.com/, doi:10.6084/m9.figshare.28795523
